# B7-H3 Chimeric Antigen Receptor Redirected T Cells Target Anaplastic Lymphoma Kinase-Positive Anaplastic Large Cell Lymphoma

**DOI:** 10.3390/cancers12123815

**Published:** 2020-12-17

**Authors:** Zhenguo Zi, Haihong Zhao, Huanyu Wang, Xiaojing Ma, Fang Wei

**Affiliations:** 1 Sheng Yushou Center of Cell Biology and Immunology, Joint International Research Laboratory of Metabolic & Developmental Sciences, School of Life Sciences and Biotechnology, Shanghai Jiao Tong University, Shanghai 200032, China; zizhenguo@sjtu.edu.cn (Z.Z.); Huanyu_Wang@sjtu.edu.cn (H.W.); xim2002@sjtu.edu.cn (X.M.); 2 Department of Obstetrics and Gynecology, The Fifth People’s Hospital of Shanghai, Fudan University, Shanghai 200240, China; zhaohaihong@5thhospital.com

**Keywords:** ALCL, ALK, B7-H3, CAR-T

## Abstract

**Simple Summary:**

Although chemotherapy is associated with high relapse rates and numerous side effects, it is still used as the front line treatment of anaplastic large cell lymphoma (ALCL). Therefore, alternative treatment options for ALCL are needed. In this study, we show that B7-H3 is a novel and promising target in ALCLs, and demonstrate that B7-H3 directed chimeric antigen receptor (CAR) T cells have therapeutic potency in controlling ALCL tumor growth.

**Abstract:**

Potent CAR-T therapies that target appropriate antigens can benefit the treatment of anaplastic lymphoma kinase-positive (ALK^+^) anaplastic large cell lymphoma (ALCL), which is the most common subtype of T cell lymphoma. In this study, we observed overexpression of B7-H3 in ALCL cell lines derived from clinical samples and differential expression of B7-H3 in an ALK-induced T cell transformation model. A B7-H3-redirected CAR based on scFv from mAb 376.96 was developed. B7-H3 CAR-T cells showed strong cytotoxicity and cytokine secretion against target ALCL cells (SUP-M2, SU-DHL-1, and Karpas 299) in vitro. Furthermore, the B7-H3 CAR-T cells exhibited proliferative capacity and a memory phenotype upon repeated antigen stimulation. We demonstrated that B7-H3 CAR-T cells could promptly eradicate ALCL in murine xenografts. Taken together, B7-H3 is a novel and promising target in ALCLs and B7-H3 CAR-T may be a viable treatment option for ALCL.

## 1. Introduction

T cells armed with CD19-redirected chimeric antigen receptor (CAR) offer an effective therapeutic option for patients with B cell lymphoid malignancies [1]. The development of CAR-T therapy has fueled research and clinical studies to search for suitable targets to treat other cancer types, including ALK^+^ ALCL [2,3].

As the most typical and well-defined ALCL type, ALK^+^ ALCL patients are generally children and young adults. ALK^+^ ALCL is the most common subtype of T cell lymphoma in children and represents 10–30% of all lymphomas [4,5]. Although the understanding of ALK^+^ ALCL biology has deepened, treatment has not improved for the past 30 years. The 2-year overall survival (OS) is over 90%, but event-free survival (EFS) is lower, ranging from 59% to 76%, depending on the treatment protocol used [3,6,7], which unfortunately means that 10% of children do not survive with an ALCL diagnosis. A larger portion of survivors suffer from intensive therapies [8]. It is reported that approximately 70% of patients with ALK^+^ ALCL relapse after initial chemotherapy [9]. Therefore, there is a clear need to search for new therapies when treating ALCL.

B7 homolog 3 (B7-H3), also named CD276, is a member of the B7 family of immune regulatory proteins [10]. Encoded by nine exons, human B7-H3 has two isoforms (2IgB7-H3 and 4IgB7-H3) [11,12]. B7-H3 protein is expressed at low levels in normal human tissues, while the overexpression of B7-H3 has been observed among several kinds of solid cancers, including prostate cancer, renal cell carcinoma, non-small cell lung cancer, and breast cancer, etc. [13]. The expression level of B7-H3 is associated with disease progression, risk of recurrence, reduced survival, and drug resistance. Therefore, B7-H3 is suggested to be a promising target for cancer immunotherapy. A number of anti-B7-H3 approaches, including antibody drug conjugated therapies, a drug targeting B7-H3 through antibody-dependent cell-mediated cytotoxicity, a bispecific antibody, CAR-T cell therapy, and a combination of therapies have been conducted in preclinical or clinical trials in different stages [14]. Among these approaches, CAR-T cells that target B7-H3 were shown to be effective on several solid tumor types (including pancreatic ductal adenocarcinoma, ovarian cancer, melanoma, glioblastoma, neuroblastoma, and a variety of pediatric cancers) [15,16,17], and hematological tumors (including extranodal nasal natural killer (NK)/T cell lymphoma and acute myeloid leukemia) both in vitro and in vivo [18,19]. Moreover, it was reported that B7-H3 CAR-Ts can significantly control tumor growth in a syngeneic tumor model and multiple preclinical trials without evident toxicity [17].

Previously, we had demonstrated that in vitro transduction of normal human CD4 T lymphocytes with nucleophosmin (NPM)-ALK could result in their malignant transformation to gain features recapitulating native ALCLs [20]. Herein, we showed that multiple membrane genes, including B7-H3, were differentially expressed in mRNA levels during the process. The upregulation of B7-H3 was confirmed in multiple ALCL cell lines. Therefore, we sought to evaluate whether B7-H3 could serve as a novel target for CAR-T therapy against ALCL.

## 2. Results

### 2.1. B7-H3 Is Overexpressed in ALK-Induced ALCL

**Figure 1:**
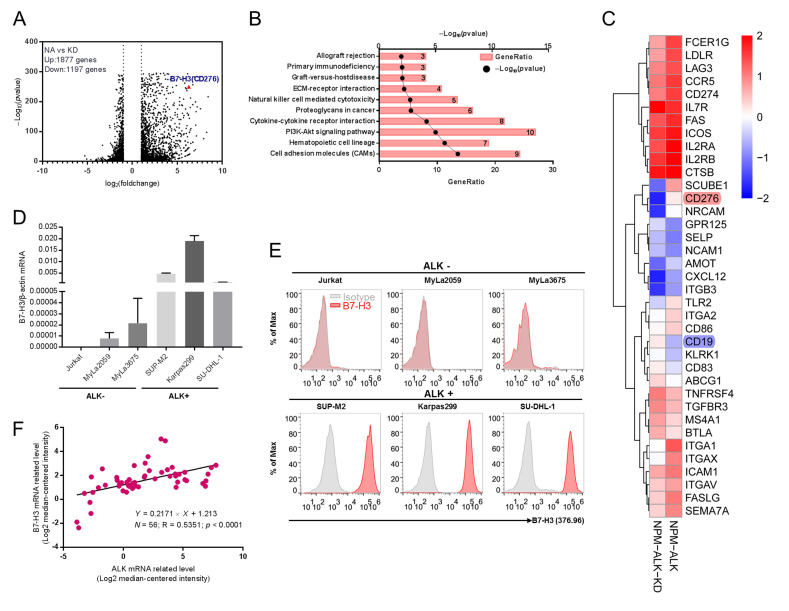
Overexpression of B7-H3 is detected in ALK^+^ ALCL. (**A**) Purified CD4+ T cells were stimulated with a T cell expander (bead-immobilized CD3 and CD28 antibodies) and either transduced with wild-type NPM-ALK (NA) or enzymatically inactive NPM-ALK mutant (KD). RNA was isolated on day 10 after transduction and deep sequencing was performed. Volcano plots depict differently expressed genes (DEGs) in NA and KD transduced CD4+ T cells. B7-H3 (CD276) is the red triangle. (**B**) Pathway enrichment analysis of different expressed membrane proteins (DEMGs) in NA vs. KD transduced CD4+ T cells. The top 10 pathways were presented. (**C**) The top 37 altered DEMGs between the NA and KD transduced CD4+ T cells group were presented in the heatmap. (**D**) B7-H3 mRNA level was analyzed by quantitative reverse-transcriptase PCR (qPCR) in both ALK-negative and ALK-positive cell lines. (**E**) B7-H3 expression on cell membrane was evaluated via flow cytometry analysis. Isotype control (grey graphs, isotype control rabbit IgG as primary antibody and anti-rabbit Fc as secondary antibody) and B7-H3 (red graphs, anti-B7-H3 antibody as primary antibody and anti Fc as secondary antibody). (**F**) B7-H3 expression positively correlates with ALK expression found on Oncomine database (*n* = 56, R = 0.5351).

Previously, we demonstrated exogenous expression of NPM-ALK (NA) but not its catalytic deactivated version (kinase dead, KD) in normal primary human CD4+ T cells could result in T cells’ malignant transformation fully recapitulating the native ALCLs [20]. Our data suggested that the transformation process of NPM-ALK could take about 1 month. To search for putative tumor-associated antigens in the early transformation phase, we similarly activated purified normal CD4^+^ T cells via CD3/CD28 beads and transduced them with lentiviral vector expressing NA or KD [20]. Then, 10 days later, when the transduction signal from the CD3/CD28 beads ceased, RNA was isolated from the samples and we performed a transcriptome deep sequencing. In total, 1197 genes were significantly downregulated, while 1877 genes were upregulated in the NPM-ALK transduced sample compared to the KD sample (Figure 1A). Differential expressed membrane genes (DEMGs) through GO analysis were sorted out to explore significantly altered signaling pathways (Figure 1B) through KEGG pathway analysis. To search for putative tumor-associated antigens suitable for chimeric antigen receptor targeting, a heatmap of DEMGs with >2-fold expression changes was plotted. Interestingly, B7-H3 was one of the most upregulated genes in NPM-ALK-transformed T cells (Figure 1C). To confirm whether B7-H3 was upregulated in ALCLs, quantitative PCR was performed to detect mRNA expression in ALCL and other T cell tumor lines (Figure 1D). The relative higher B7-H3 mRNA was observed in ALCL cell lines SUP-M2, Karpas299, and SU-DHL-1, but not in other T cell lines. To further confirm B7-H3′s expression, cells were stained with an anti-B7-H3 antibody and evaluated via flow cytometry. Consistent with the mRNA level, a high level B7-H3 protein was observed in cell lines SUP-M2, Karpas299, and SU-DHL-1, but not in Jurkat, Myla2059, and Myla3675 cells both by surface (Figure 1E) and intracellular staining (Figure S1A). To further evaluate the expression pattern of B7-H3 in more clinically relevant samples, we analyzed the mRNA level of ALK and B7-H3 in a total 56 ALCL patients and cell lines collected (34 ALK^+^ and 22 ALK^−^ ALCL) via the Oncomine database [21]. The data showed a strong positive correlation between ALK and B7-H3 expression (Figure 1F and Figure S1B). Together, these results indicated that B7-H3 might serve as a putative target to develop CAR-T therapy for ALCLs.

### 2.2. B7-H3-Redirected CAR-T Cells Have Similar Growth Rate as CD19-CAR-T Cells In Vitro

**Figure 2:**
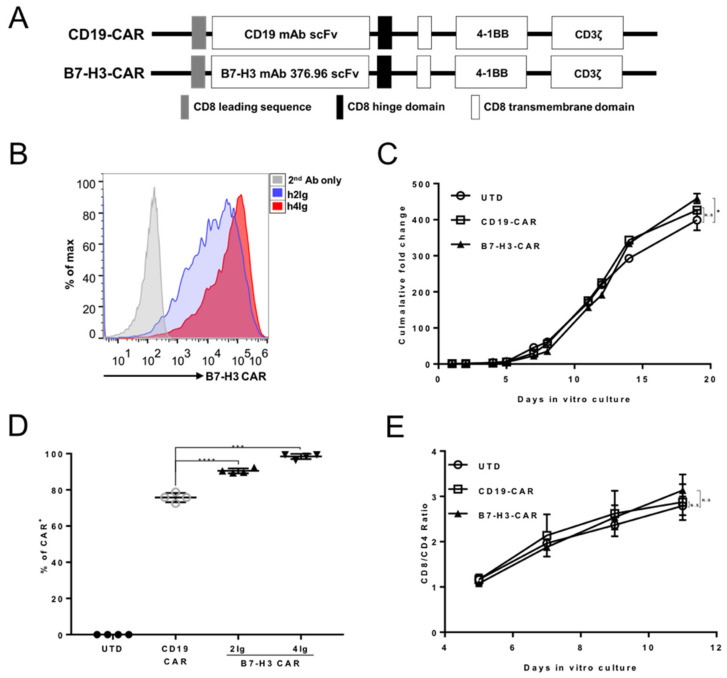
Generation and validation of B7-H3 CAR. (**A**) Schematic representation of the B7-H3 CAR. (**B**) The expression of B7-H3 CAR in T cells was evaluated via h2Ig or h4Ig antigens staining (h2Ig shown in blue, h4Ig shown in red). Secondary antibody-only staining served as the control (shown in grey). (**C**) Expansion kinetics of UTD, CD19, and B7-H3 CAR-T cells in vitro (*n* = 5). Error bars denote SD (* *p* = 0.0358, no significant difference showed as N.S). (**D**) Summary of the CD19 and B7-H3 CAR-T transduction efficiency (*n* = 4). The horizontal bars represent the mean values. Error bars denote SD (*** *p* < 0.001, **** *p* < 0.0001). (**E**) The CD8/CD4 ratio of in vitro culturing of UTD, CD19, and B7-H3 CAR-T cells at indicated days detected by fluorescence-activated cell sorting (FACS) staining.

Next, we sought to construct B7-H3-redirected CAR in a lentiviral vector that encoded an anti-B7-H3 mAb 376.96 scFv fragment, a 4-1BB costimulatory domain, and a CD3-ζ signaling domain (Figure 2A). CD19 scFv was constructed into the same backbone to serve as the control. The expression of B7-H3 CAR in the human primary T cells was confirmed by the staining of either B7-H3 hIg2 or hIg4 isoforms. (Figure 2B). More hIg4 than hIg2 staining positive cells were observed, which is consistent with a previous report that B7-H3 CAR shows a higher affinity to hIg4 than hIg2 [17]. The B7-H3 CAR-T cells showed similar expansion capacity as CD19 CAR-T and un-transduced T cells (UTD) (Figure 2C). In addition, B7-H3 CAR lentiviruses exhibited efficient infection, suggesting that the B7-H3 CAR-T cells might be easier for industry production (Figure 2D). The CD8/CD4 ratio was increased for all groups including B7-H3 CAR-T with the addition of IL-2 (Figure 2E), which is consistent with a previous report that IL-2 can reduce the minimal threshold of TCR signaling required for CD8 T cell proliferation; however, the threshold for CD4 T cell proliferation in vitro involves differential STAT5 phosphorylation [22].

### 2.3. B7-H3 Redirected CAR-T Cells Show Their Potency in Controlling ALCLs In Vitro

**Figure 3:**
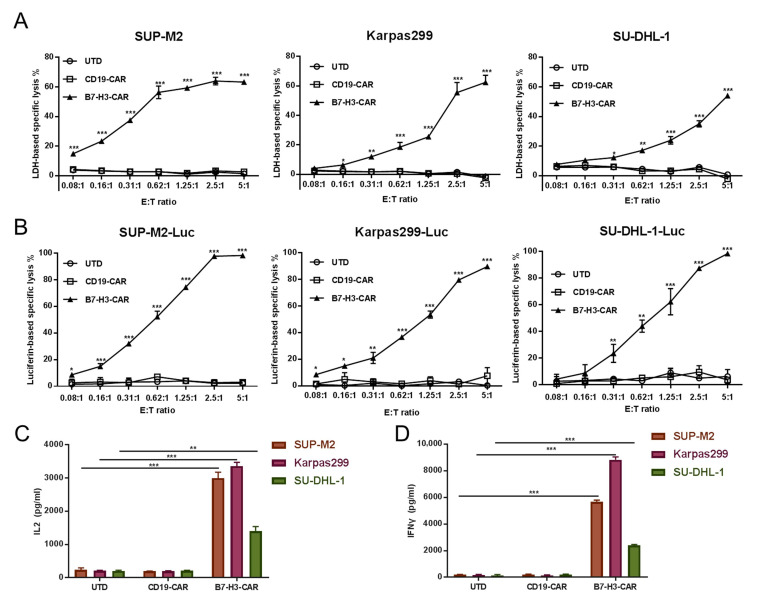
B7-H3 T cell effects on B7-H3-expressing ALCL cells. CD19 CAR-T and B7-H3 CAR-T cells were normalized to the same expression efficiency. ALK^+^ lymphoma cell lines (**A**) or their luciferase derivatives (**B**) were co-cultured with UTD, CD19 CAR-T, or B7-H3 CAR-T cells in the indicated E: T ratio for 6 h. (**A**) The percentage (%) release of LDH was calculated as killing efficiency of the effector T cells. (**B**) The percentage (%) activity of luciferase from SUP-M2-Luc, Karpas299-Luc, and SU-DHL-1-Luc was calculated as the killing efficiency of effector T cells. Next, (**C**,**D**) effector T cells (UTD, CD19 CAR-T, or B7-H3 CAR-T) and target cells (SUP-M2, Karpas299, or SU-DHL-1) were co-cultured at a 1:1 ratio for 6 h, then the supernatants were collected. IL-2 (**C**) and IFN-γ (**D**) levels were measured via ELISA. All error bars represent SD (* *p* < 0.05, ** *p* < 0.01, *** *p* < 0.001).

We chose to measure B7-H3 CAR-T cells’ effector function in its cytotoxicity and cytokine production ability. ALCL cell lines SUP-M2, Karpas299, and SU-DHL-1 were chosen as targets. Cytotoxicity was measured using two different approaches—the LDH release cytotoxic assay and luciferase-based assay in various E:T ratios. Increased cytotoxicity along with an increased E:T ratio was observed in B7-H3 CAR-T cells to target ALCL cell lines in both approaches. Furthermore, >60% specific lysis was reached when the E:T ratio was 5:1 for all targets in the LDH release cytotoxic assay (Figure 3A). No or very low cytotoxicity effects of UTD or CD19 CAR-T cells were observed. In contrast, almost neglected cytotoxicity of B7-H3 CAR-T was observed in Jurkat cells (Figure S2). To confirm this effect, we generated stable expressing luciferase cell lines from parental SUP-M2, Karpas299, and SU-DHL-1 cell lines as well as the performed luciferase-based assay. This was consistent with data from the LDH release cytotoxic assay, and B7-H3 CAR-T cells showed their potency in controlling SUP-M2, Karpas299, and SU-DHL-1 (Figure 3B). We also evaluated the cytokine production activity of B7-H3 CAR-T cells when co-cultured with ALCLs. The secreting of IL-2 and IFNγ was observed at a high level when B7-H3 T cells were co-cultured with ALCLs. In contrast, UTD and CD19 CAR-T control cells showed minimal secretion of both cytokines. Interestingly, Karpas299 could stimulate B7-H3 CAR-T to produce a higher amount of IL-2 and IFN-γ when compared with SU-DHL-1, which was positively correlated with ALCL total B7-H3 expression levels.

### 2.4. B7-H3 Redirected CAR-T Cells Can Be Expanded upon Repeat Antigen Stimulations

**Figure 4:**
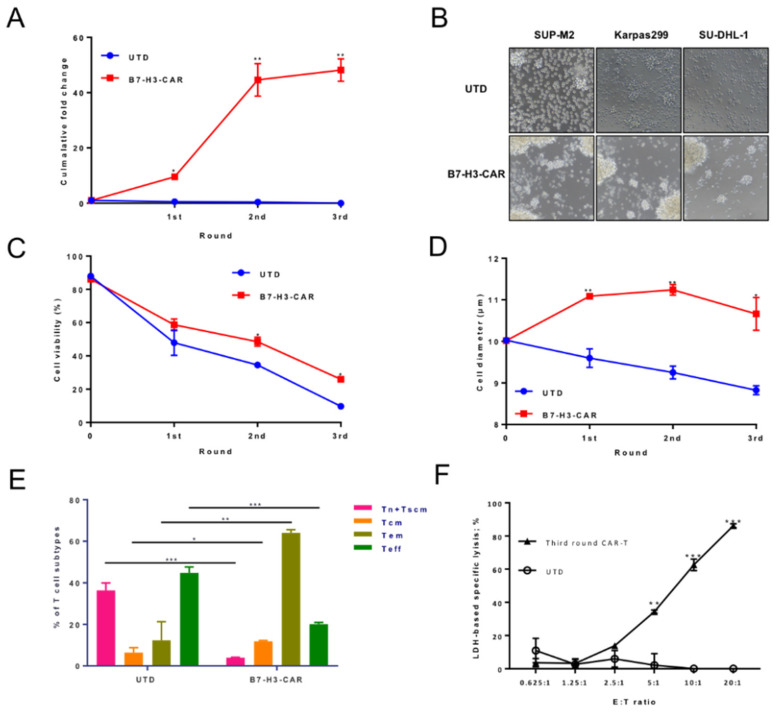
The expansion of B7-H3 redirected CAR-T cells upon repeat antigen stimulations. (**A**) B7-H3 CAR-T cells were co-cultured with SUP-M2 cell lines at an E:T ratio of 1:1. After every 3 days, CAR-T cells were counted by CD3 FACS staining, and the same number of CAR-T cells were re-plated on a new batch of SUP-M2 cells. The process was repeated for two more stimulations. Cumulative fold expansion of CAR-T cells in each stimulation time point is shown while UTD is the control. (**B**) Representative microscope images (100×) of UTD (up) and B7-H3 CAR-T (down) co-cultured with SUP-M2, Karpas299, and SU-DHL-1 at a 1:1 ratio after the first round. (**C**) Cell viabilities in a repeat antigen stimulation assay were measured using trypan blue staining at each time point. (**D**) Cell diameters in a repeat antigen stimulation assay were measured via a cell counter (CounterStar) at each time point. (**E**) After the second stimulation, the phenotypes of T cells were flow cytometric quantified based on CD45RA and CD62L staining. The gates were set for effect memory T cells (T_em_, CD45RA−CD62L−), central memory T cells (T_cm_, CD45RA−CD62L+), naïve T cells (CD45RA+CD62L+), and T effector cells (T_eff_, CD45RA+CD62L−) [23] in UTD and CAR-T cells (*n* = 3). Error bars denoted SD. Two-way ANOVA with a t test adjusted *p* value was calculated between UTD and B7-H3 CAR-T cells (* *p* < 0.05, ** *p* < 0.01, *** *p* < 0.001). (**F**) The cytotoxicity of B7-H3 CAR-T cells after three rounds of re-stimulation was measured via the LDH assay when co-cultured with SUP-M2 cells at the indicated E: T ratio. In vitro cultured unstimulated UTD served as the control.

The expansion of CAR-T cells in patients is critical in determining the clinical outcome. In vivo, CAR-T cells are required to expand in the setting of continuous exposure to target antigens in order to eradicate large tumor burdens. Therefore, it would be useful to evaluate the expansion ability of B7-H3 CAR-T cells when they meet with repeat antigens expressed on the ALCL surface, which is mimicked by the fresh added ALCL cells for multiple rounds (Figure 4A). During each round of stimulation, CD3 was used as a distinguishing marker for tumor cells and T cells, and the E: T ratio was measured before and after each round of stimulation (Figure S3). The cumulative fold changing data acquired after each stimulation revealed the substantial differences in the expansion between UTD and B7-H3 CAR-T cells. After three rounds of stimulation, the B7-H3 CAR-T cells could expand ~50-fold. The CAR-T cells could besiege ALCL cells and rapidly expand as massive colonies (Figure 4B), as shown by microscopy. However, cell viability gradually decreased in both groups after each round of stimulation, while B7-H3 CAR-T cells showed a higher survival rate compared with the UTD group (Figure 4C). Meanwhile, B7-H3 CAR-T cells maintained a relatively larger size, which indicated their active stages (Figure 4D). In addition, T cell phonotypes were analyzed after the second round of stimulation. Most B7-H3 CAR-T cells showed a central memory or effect memory phenotypes. A small population of cells was either naïve/stem cell memory or effector phenotypes. (Figure 4E). These also support B7-H3 CAR-T cells, which can prompt a response in eradicating tumor cells and their expansion ability. In addition, we performed the LDH-based killing assay using third round stimulated T cells co-cultured with tumor cells in different ratios. Furthermore, >90% specific lysis was also reached in B7-H3 CAR-T cells with a three-round antigen-specific stimulation when the E:T ratio was 20:1 (Figure 4F), though only less than half of the activity was maintained at E:T = 5:1 when compared to B7-H3 CAR-T cells without re-stimulation. Together, these results indicated that B7-H3 CAR-T cells could be persistently activated when antigens were presented.

### 2.5. B7-H3 Redirected CAR-T Cells Show Potent Antitumor Effect In Vivo

**Figure 5:**
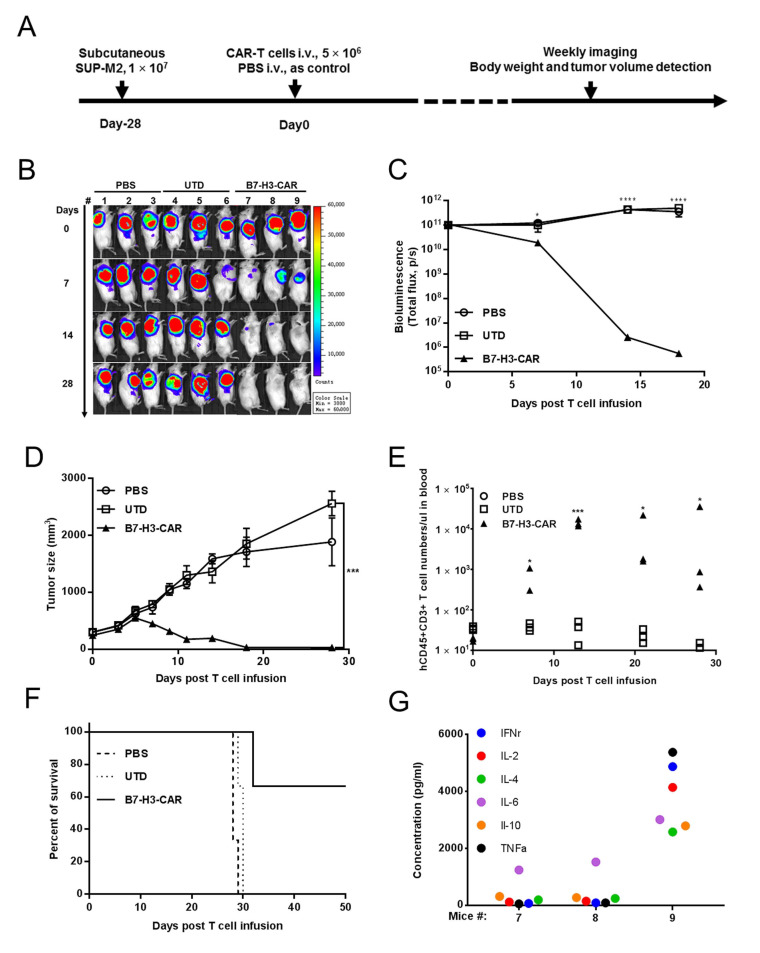
B7-H3 CAR-T cells can control ALCL growth in vivo. (**A**) The schematic diagram shows the workflow of the SUP-M2 xenograft model infused with B7-H3 CAR or control (UTD) T cells. (**B**) Tumor killing capacity of B7-H3 CAR-T cells in NOG mice with a SUP-M2-Luc xenograft model. (**C**) Bioluminescence kinetics of SUP-M2-Luc tumor growth (3 mice/group) in the xenograft model. (**D**) Tumor size was measured via a Vernier caliper. (**E**) The absolute cell counts of human T lymphocytes in mice blood after T cell infusion were quantified by hCD45hCD3 staining every week. (**F**) The Kaplan–Meier survival curve of mice in the SUP-M2-Luc xenograft model. Mice were sacrificed when the tumor volume reached 2000 mm^3^. The Log-rank test *p* value of UTD versus B7-H3 CAR-T (5 × 10^6^ cells/mouse) was 0.0295. (**G**) Multiple cytokines (IFNγ, IL-2, IL-4, IL-6, IL-10, and TNFα) levels detected by the Multiplex Cytokine Assay were elevated in the serum of B7-H3 CAR-T-treated mice at day 28 (#7 and #8 were alive; #9 died at day 32).

We subsequently sought to evaluate B7-H3 CAR-T cells’ effect in controlling ALCL in vivo during the xenograft NOG mouse model, which utilized SUP-M2 cells that stably expressed luciferase (Figure 5A). Our pilot experiments (Figure S4A) showed that B7-H3 CAR-T cells could effectively eradicate tumors in 14 days. Moreover, no tumor was established in B7-H3 CAR-T injected mice when re-challenged by the same tumor cells at day 21. On the contrary, the tumors kept growing after treated with either UTD or CD19-CAR-T with no statistically different rate (Figure S4B,C). The controlling of B7-H3 CAR-T cells to SUP-M2 cells was further monitored by tumor burden measurements (bioluminescence imaging, BLI, and tumor size), T cell persistence (hCD45^+^CD3^+^ cell counts in blood), body weight, and survival rate. A single dose (5 × 10^6^ per mouse) of B7-H3 CAR- or UTD T cells was injected into mice with a pre-established tumor burden. B7-H3 CAR-T cells showed a prompt and potent antitumor effect within 7 days of infusion (Figure 5B). It initially decreased in tumor BLI at day 7, further at day 14, and eventually to basal level at day 28 in all three mice (Figure 5C). The rapid reduction in tumor burden was also confirmed by tumor size measurement (Figure 5D). The scFv fragments were also detected at day 7 to confirm the expansion of CAR-T cells (Figure S4D). Compared with the control groups, no obvious weight change was observed for the B7-H3 CAR-T treatment groups (*p* < 0.05; Figure S4E). The exponential expansion of B7-H3 CAR-T cells was observed after the first two weeks in mice post infusion. T cell growth stopped at day 14 in two out of three mice when the tumor burden was low, while it kept expanding in one exceptional mouse (Figure 5E). All mice in the PBS and UTD groups died around 30 days, and tumor burden free survival was maintained in two out of three mice (T cells stopped further expansion) for at least 50 days. The exceptional mouse in the B7-H3 CAR-T treatment group (T cells kept further expansion) swiftly lost weight and died at day 32 (Figure 5F). Blood was drawn from this mouse on day 30 to detect T cell abundance. Astonishingly, tremendous human origin T cells were observed in this mouse with 96.66% peripheral blood mononuclear cells (PBMCs) shown as hCD45+ (Figure S4F). The cytokines (IFNγ, IL-2, IL-4, IL-6, IL-10, and TNFα) were profiled in the serum of three mice in the B7-H3 CAR-T group in Figure 5G, suggesting that a cytokine storm might result in the death of this mouse. Through autopsy, we observe that the mouse’s liver had a sign of necrosis and its spleen was enlarged. No other tissue damage was observed. (Figure S4G). Massive lymphocytic infiltration in the lung was observed via hematoxylin and eosin staining, while the cellular morphology of other tissues was presented as normal (Figure S4H).

Taken together, our study demonstrates that B7-H3 is a potential target for treating ALCLs and provides evidence for the application of B7-H3 CAR-T in treating ALCLs.

## 3. Discussion

Although the overall survival (OS) rate in treating ALCL patients is as high as 90%, the event-free survival (EFS) rate is only at an acceptable level. The regimen of chemotherapy is still used to this day [8]. Reducing the side-effects by supplying chemotherapy with other potential therapeutic targets and treatments is attractive for ALCL research. Here, we show that B7-H3 is upregulated in an ALK-induced T cell transformation model and ALCL cell lines, proving that B7-H3-directed CAR-T cells have potency when controlling ALCL tumor growth in vitro and in vivo.

Appropriate tumor-associated antigens are the first challenge when developing safe and effective CAR-T-based therapies. An ALK-driven T cell transformation model developed previously could allow us to study early genetic changes in tumorigenesis. Our data revealed that B7-H3 could be an early event associated with tumorigenesis. Mechanisms for the correlation between B7-H3 and tumor progression have been explored from both immunological and non-immunological aspects [13]. The expression of B7-H3 is associated with worse clinical outcome. The inhibitory and stimulating abilities of B7-H3 towards the proliferation of T cells (both CD4+ and CD8+) have been described previously [11,24,25]. Whether B7-H3 upregulation promotes tumor growth needs to be further analyzed. Thus far, there is no conclusive statement regarding the function of B7-H3, and our model might provide a simple system to address it.

The potency and persistency of adoptive transfer cells are critical for the CAR-T-based therapy. A similar potency level of B7-H3 CAR-T cells was shown in ALCL models as in other models described elsewhere [17]. The growth of an ALCL tumor was controlled 7 days after T cell infusion and a complete response was observed at close time points, as was the case in other models [17]. It has been reported that 40–60% patients in CD19 CAR-T clinical trials failed due to poor T cells persistence or the emergence of CD19-negative clones [26]. The internal signal domain chosen in this study was a 4-1BB signaling domain. Although it is has been shown that B7-H3.CAR-28ζ-Ts and B7-H3.CAR-BBζ-Ts showed equivalent antitumor properties in tumor models [17], incorporating 4-1BB signaling into the CAR molecular cellular domain could enhance CAR-T cell expansion in vivo in certain models [27]. This might account for the persistence of B7-H3 CAR-T cells upon repeated antigen stimulation. B7-H3 CAR-T cells persisted for up to 30 days post infusion in our xenograft model, which was consistent with other observations.

The safety issue of CAR-T cells also needs to be taken into account. It was shown previously that B7-H3 CAR-T cell efficacy largely depends upon surface target antigen density on tumor tissues [28]. We only observed very low effector activity when B7-H3 low-expressing Jurkat cells were the target. This disparity of cytotoxicity of B7-H3 CAR in B7-H3 density provided safety in clinical application. Consistent with PDAC xenograft models [17], no toxicity issue was observed in our prostate, liver, gastric, and glioma tumor models (data not shown). However, we observed that CAR-T cells caused death in one mouse at day 30 after T cell infusion. Massive human T cells peripherally existed, demonstrating that CAR-T cells had an uncontrolled expansion in mice even after ALCL tumors vanished. B7-H3 CAR-T cells have shown more safety and less toxicity when treating malignant tumors in the nervous system and pancreatic ductal adenocarcinoma [17,29]; however, the safety of B7-H3 CAR-T cells has been proven in other models only at the 2 week window post T cell infusion [17]. Our data suggested that it is worthwhile monitoring CAR-T cell counts to better evaluate the potentially lethal toxic effects of B7-H3 CAR-T therapy, even in other tumor types. Furthermore, tightly controlled cytokine release syndrome and neurologic toxicities induced by CAR-T through tocilizumab or corticosteroids administration can be clinically applied [30].

## 4. Materials and Methods

### 4.1. Cell Isolation, Culturing, Gene Transferring, and Expansion

For the use of blood samples from healthy donors, written informed consent was obtained in accordance with the approval of the Shanghai Jiao Tong Human Sample Ethical Committee on 1 March 2014 (2014-001). Peripheral blood mononuclear cells (PBMCs) were purified by the EasySep™ Human T Cell Isolation Kit (STEMCELL, Seattle, WA, USA) and cultured in X-VIVO medium (Lonza, Alpharetta, GA, USA) supplied with 100 U/mL IL-2 (R&D system, Minneapolis, MN, USA). T cells were expanded and transduced as previously described by Wei et al. [31]. Tumor cell lines Jurkat, MyLa2059, MyLa3675, SUP-M2, Karpas299, and SU-DHL-1 were kindly provided by Dr. Mariusz Wasik (University of Pennsylvania, Philadelphia, PA, USA). SUP-M2-Luc, Karpas299-Luc, and SU-DHL-1-Luc cell lines were generated by the transduction of lentivirus-containing DNA encoding both red fluorescent protein (RFP) and luciferase and fluorescence-activated cell sorting (FACS) based on RFP expression. All cells were maintained in RPMI 1640 culture medium, supplied with 10% fetal bovine serum and 50 U/mL Penicillin-Streptomycin (Thermo Fisher Scientific, WS, MA, USA). All cells were maintained in a 5% CO_2_ incubator at 37 °C.

### 4.2. Plasmids

The DNA fragment encoding B7-H3-specific scFv derived from mAb 376.96 was synthesized and fused to the DNA fragment encoded 4-1BB and CD3ζ signaling domains, then subcloned into lentivirus vector pCDH. CD19 CAR control was constructed by substituting B7-H3 scFv sequence with CD19 scFv.

### 4.3. Antibody Staining and Flow Cytometry

Surface staining was performed following the manufacturer’s instructions. The following antibodies were used for cell surface staining: APC-CD45, BV421-CD3, APC-CD4, PE-CD8, FITC-CD45RA, BV605-CD62L (CD45 and CD3 antibodies were purchased from Biolegend, San Diego, CA, USA, the others were from BD Biosciences, San Jose, NJ, USA). Human B7-H3 in tumor cell lines were stained by either surface or intracellular (pre-fixed and permeabilized by the Fixation/Permeabilization Solution Kit) staining by the anti-B7-H3 antibody (Abcam, Camb, MA, USA), followed by the PE-conjugated donkey anti-rabbit IgG antibody. The transduction efficiency of CAR-T cells was analyzed using 4Ig-hB7-H3 or 2Ig-hB7-H3 protein with mFc tag (ACRO, Beijing, China), followed by FITC-conjugated rabbit anti-mouse Fc antibody (Biolegend, San Diego, CA, USA) staining. Data were acquired with Attune NxT (Thermo Fisher Scientific, WS, MA, USA) and analyzed by FlowJo software v.10.0.7.

### 4.4. Quantitative Real Time PCR

Total RNA was isolated by Trizol reagent and reverse transcribed into cDNA using Hifair^®^ Ш 1st Strand cDNA Synthesis SuperMix (Yeasen, Shanghai, China). Real-time PCRs were performed using CFX (Bio-Rad, Hercules, CA, USA) in triplicate. The primers (Forward: 5-CTGGCTTTCGTGTGCTGGAGAA-3; Reverse: 5- GCTGTCAGAGTGTTTCAGAGGC-3) were used to measure the B7-H3 mRNA level in the real-time PCR (RT-PCR) assay.

### 4.5. ELISA

B7-H3 CAR-T cells and tumor cells were co-cultured in X-VIVO medium in 24-well plates at 37 °C for 18 h, then the supernatant was collected and the ELISA assay was performed to measure IL-2 and IFNγ secretion levels following the instruction manual of ELISA kits (BD Biosciences, San Jose, CA, USA). The ELISA data calculation formula used was: Concentration = Experimental (OD450) × dilution factor based on the standard curve

### 4.6. Cytotoxicity Assays

CAR-T cells and tumor cells were co-cultured at an inclined set of the E: T ratio for 6 h in X-VIVO medium. Cytotoxicity was evaluated by two independent approaches. The LDH release assay was performed following the instruction of the CytoTox 96^®^ Non-Radioactive Cytotoxicity Assay kit (Promega, Madison, WI, USA). Briefly, the supernatants were collected and incubated with the CytoTox 96 Reagent for 30 min, and the absorbance value of each sample (OD490) was reached by Multiscan Spectrum (Molecular devices, San Jose, CA, USA). Percentage of specific lysis was calculated by the following formula: (test release – spontaneous release)/(maximal release − spontaneous release) × 100. The luciferase report assay was performed following the instructions of the Steady-Glo^®^ Luciferase Assay System (Promega, Madison, WI, USA). The same volume of Steady-Glo reagent was added into the cell mixture and the mix was incubated for 5 min at room temperature, then luminescence values were obtained by Multiscan Spectrum (Molecular devices, San Jose, CA, USA). Percentage of specific lysis was calculated by the following formula: 1 – (maximal signal – test signal) × 100.

### 4.7. Repeat Antigen Stimulation Expansion Assay

B7-H3 CAR-T cells (1 × 10^5^) and SUP-M2 cells (1 × 10^5^) were suspended in 2 mL X-VIVO medium, then co-cultured in 6-well plates at 37 °C without the addition of IL2, following a protocol described elsewhere [32]. At day 3 (recorded as the first stimulation), cell number, diameter, and viability were detected by a cell counter (Countstar, Shanghai, China). T cells were then washed with PBS twice and the stimulation with fresh SUP-M2 cells was repeated; the SUP-M2: T cell ratio was confirmed through FACS staining before and after the rounds of stimulation. Three days later (recorded as the second stimulation), the same dataset was acquired. In addition, T cell phenotype was determined by flow cytometry. Then, the rest of the cells were stimulated for the third time with fresh SUP-M2 cells with the same procedure as the previous stimulation. Cumulative fold expansion is determined by [(Fold expansion_n_ × Fold expansion_n+1_) …].

### 4.8. Xenograft Assay

Six to 8-week-old immuno-deficient NOD.Cg-Prkdc^scid^Il2rg^tm1Sug^ (NOG) female mice were purchased from Vital River Laboratory Animal Technology Co., Ltd, BJ, CHN. Mice were maintained under specific pathogen-free conditions, and all procedures were handled under the instruction of the requirements of the National Institutes of Health and Institutional Animal Care and Use Committee. The animal experiments were approved by the Shanghai Jiao Tong University Animal Care Committee (A2019012). Each mouse was injected subcutaneously behind the right foreleg with 1 × 10^7^ SUP-M2-Luc (with 50% Matrigel purchased from Corning Incorporated, Corning, NY, USA). On day 28, when the tumor volumes reached 200 mm^3^, the mice were randomly divided into three groups, three mice per group. The mice were injected intravenously with 5 × 10^6^ B7-H3 CAR-T cells or un-transduced T (UTD) cells in 200 µL or equal volume of PBS, respectively. Tumor growth was monitored by a Vernier caliper every two or three days or by bioluminescence signal every week. Tumor volume was calculated as follows: tumor size = long diameter × (short diameter^2^)/2. Mice body weight was measured every two or three days for 50 days after CAR-T treatment. Blood was drawn from the mice orbit every week after CAR-T engraftment to quantify the persistency of circulating CD45+CD3+ human T cells.

### 4.9. Multiple Cytokine Assay

Blood samples were obtained from B7-H3 CAR-T-treated mice through orbit, then centrifuged at 16,000× *g* for 5 min to separate upper serum. Multiple cytokines were detected by the Human Premixed Multi-Analyte Kit (R&D system, Minneapolis, MN, USA) according to the manufacturer’s instructions. Briefly, 50 µL diluted standard or samples were added to a 96 well-plate, then mixed with a 50 µL microparticle cocktail containing IFNγ, IL-2, IL-4, IL-6, IL-10, and TNFα antibodies, incubated for 2 h at room temperature on an orbital shaker. The microparticles were then washed and incubated with Biotin-Antibody Cocktail followed by diluted Streptavidin-PE. The signals were detected by the Bio-Plex analyzer (Bio-Rad, Hercules, CA, USA), and serum cytokine levels were calculated according to standard curves.

### 4.10. RNA Sequencing, Bioinformatics and Statistical Analysis

Human T cells were transduced by lentivirus-encoded NPM-ALK WT or Kinase Dead (KD). Ten days later, total RNA were isolated from these cells and the RNA were deep sequenced by BGI (Huada Genomics Institute Co. Ltd., Shenzhen, China). The differentially expressed genes (DEGs) were selected by fold change ≥2 and false discovery rate (FDR) ≤ 0.1%. The differentially expressed membrane genes (DEMGs) were selected by GO analysis. Functional analyses of DEMGs were performed using DAVID and KEGG for ingenuity canonical pathway enrichment, while the top 10 enriched terms are presented as bar graphs.

The graphs and data analysis were generated using R software v4.0.1 and GraphPad Prism software v.7.04. Each experiment presented was repeated at least three times. Data are presented as the means ± SD. Two tailed *p* value calculation was used to measure differences between two groups. Two-way ANOVA was used to determine statistically significant differences between multiple groups, accordingly. *p*-values are represented as * (*p* < 0.05), ** (*p* < 0.01), *** (*p* < 0.001), **** (*p* < 0.0001), and NS (not significant).

## 5. Conclusions

Overall, we revealed here that the overexpression of B7-H3 is associated with ALK-induced T cell malignant transformation. This phenomenon was also observed in other ALK+ ALCL cell lines. B7-H3 CAR-T cells showed strong cytotoxicity and cytokine secretion against ALCL cells (SUP-M2, SU-DHL-1, and Karpas 299) in vitro. These CAR-T cells were presented with a hyper-proliferative feature upon repeated antigen stimulation. Moreover, B7-H3-redirected CAR-T cells can effectively eradicate an ALCL murine xenograft. Our study proves that B7-H3 is a novel and promising target in ALCLs and B7-H3 CAR-T may be a viable option in the clinical treatment of ALCL.

## Supplementary Materials

The following are available online at https://www.mdpi.com/2072-6694/12/12/3815/s1, Figure S1: The expression of B7-H3 in T cell lymphoma cell lines and ALCL clinical cases; Figure S2: B7-H3 T cell effects on B7-H3-expressing Jurkat cells; Figure S3: FACS staining of CD3 expression before and after each round of stimulation; Figure S4: In vivo model of B7-H3 CAR-T function, occupancy and autopsy in mouse.
