# Supplementary material for: B7-H3 Chimeric Antigen Receptor Redirected T Cells Target Anaplastic Lymphoma Kinase-Positive Anaplastic Large Cell Lymphoma
Source: Cancers (Basel). 2020 Dec 17;12(12):3815. doi: 10.3390/cancers12123815 (PMC7766167; doi:10.3390/cancers12123815)
Supplement: Supplementary file 1 [file cancers-12-03815-s001.pdf]

cancers

MDPI

Supplementary Materials: B7-H3 Chimeric Antigen Receptor Redirected T Cells Target Anaplastic Lymphoma Kinase-Positive Anaplastic Large Cell Lymphoma

Zhenguo Zi, Haihong Zhao, Huanyu Wang, Xiaojing Ma and Fang Wei

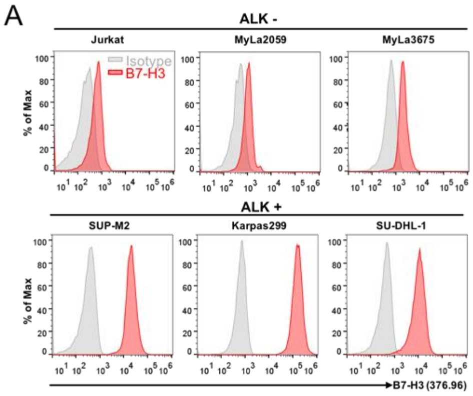

B

Oncomine lymphoma databsase	ALCL cases	Reference
Eckerle lymphoma	12	Leukemia 2009/11/01
Iqbal lymphoma	30	Blood 2010/02/04
Piccaluga Lymphoma	6	J Clin Invest 2006/12/01
Bittner Lymphoma	2	Oncogene 2006/03/09
Barretina CellLine	6	Nature 2012/03/28

Figure S1. The expression of B7-H3 in T cell lymphoma cell lines and ALCL clinical cases. A. Intracellular B7-H3 protein level was evaluated by flow cytometry analysis; B. The ALCL cases of total lymphoma database in Oncomine and related references.

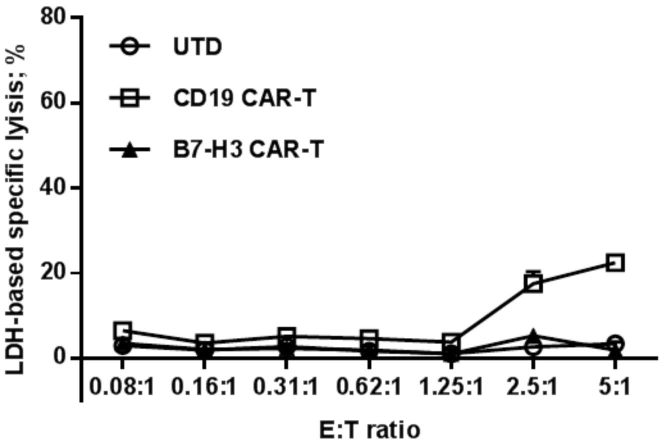

Figure S2. B7-H3 T cell effects on B7-H3-expressing Jurkat cells. CD19 CAR-T and B7-H3 CAR-T cells were normalized to the same expression efficiency. Jurkat cells were co-cultured with UTD, CD19 CAR-T or B7-H3 CAR-T cells in indicated E: T ratios for 6 hours. The percentage (%) release of LDH was calculated as killing efficiency of effector T cells.

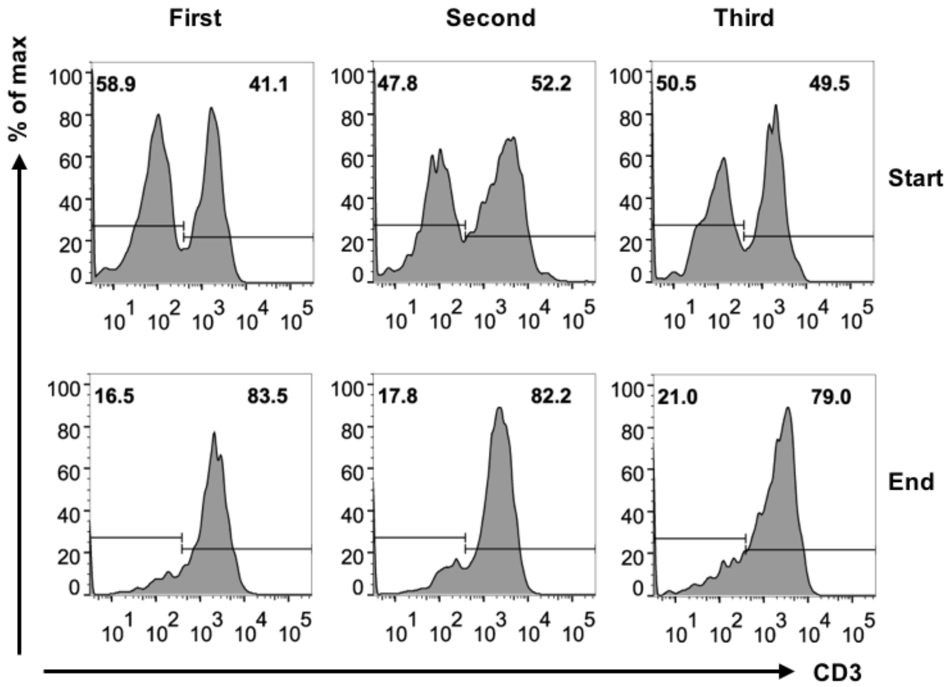

Figure S3. FACS staining of CD3 expression before and after each round of stimulation. B7-H3 CAR-T cells were co-cultured with SUP-M2 cell lines at an E: T ratio of 1:1. After every 3 days, CAR-T cells were counted by CD3 FACS staining, and the same number of CAR-T cells are re-plated on a new batch of SUP-M2 cells. The process was repeated for 2 more stimulations.

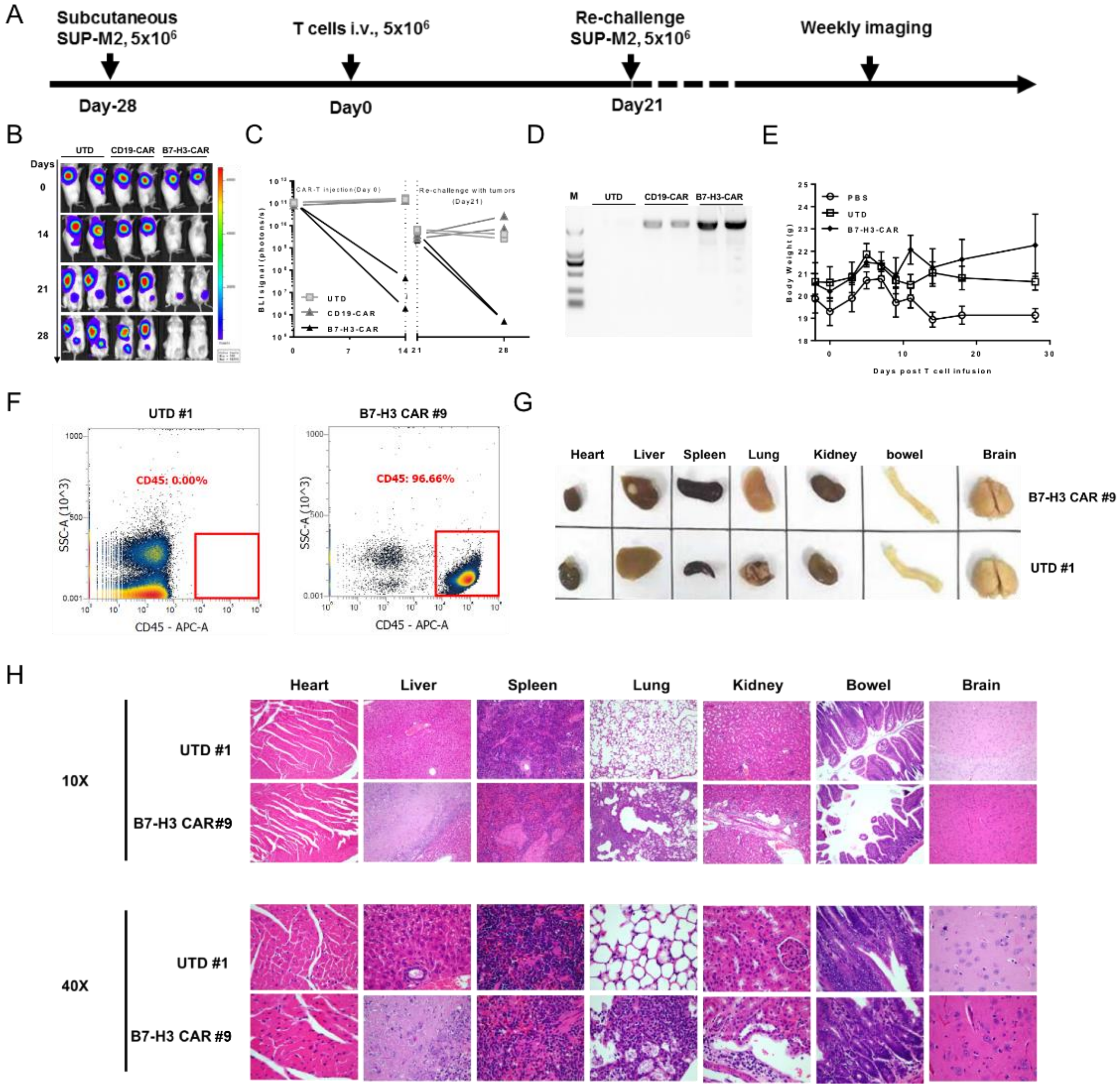

Figure S4. In vivo model of B7-H3 CAR-T function, occupancy and autopsy in mouse. (**A**) The schematic diagram shows the workflow of the SUP-M2 xenograft re-challenge model in which mice were re-challenged with tumor cells at days 21 after infused with B7-H3 CAR-T, CD19 T and UTD cells. (**B**) Tumor killing capacity of B7-H3 CAR T cells in NOG mice with a SUP-M2-Luc xenograft model shown in A. (**C**) Bioluminescence kinetics of SUP-M2-Luc tumor growth in the re-challenge model shown in (**A**). (**D**) Genome CAR elements of infused T cells were monitored by PCR in blood sample through orbit after T cells administration on day 7. (**E**) Body weight of mice in Figure 5 were measured every two or three days after PBS, UTD and B7-H3 CAR-T cell infusion (3 mice/group). (**F**) The phenotyping of PBMCs collected from mouse #9 in Figure 5 which was infused with B7-H3 CAR T cells for 30 days. Human T lymphocytes were gated by hCD45. (**G**) Autopsy of mouse #9 (in Figure 5) multiple tissues including heart, liver spleen, lung, kidney, bowel and brain. (**H**) Hematoxylin and eosin staining of autopsy collected tissues of mouse #9 (in Figure 5).

Publisher's Note: MDPI stays neutral with regard to jurisdictional claims in published maps and institutional affiliations.

© 2020 by the authors. Licensee MDPI, Basel, Switzerland. This article is an open access article distributed under the terms and conditions of the Creative Commons Attribution (CC BY) license (http://creativecommons.org/licenses/by/4.0/).
